# Hepatitis C virus can induce gene expression changes associated with hepatocarcinogenesis

**DOI:** 10.1016/j.jhepr.2026.101897

**Published:** 2026-05-29

**Authors:** Tuyana Boldanova, Fredrik Trulsson, Fahim Ebrahimi, Andrej Benjak, Matthias S. Matter, Aleksei Suslov, Stefan Wieland, Charlotte K.Y. Ng, Markus H. Heim

**Affiliations:** 1 Hepatology Research Group, Department of Biomedicine, University of Basel, Switzerland; 2 Center for Scientific Computing (sciCORE), University of Basel, Switzerland; 3 University Digestive Health Care Center Basel – Clarunis, Basel, Switzerland; 4 Institute of Pathology, University Hospital Basel, Basel, Switzerland; 5 Department for BioMedical Research (DBMR), University of Bern, Bern, Switzerland; 6 IRCCS Humanitas Research Hospital, Rozzano, Milan, Italy; 7 Department of Biomedical Sciences, Humanitas University, Pieve Emanuele, Milan, Italy

**Keywords:** Viral oncogenesis, liver cancer, predictive signature, molecular imprinting, pre-neoplastic alterations

## Abstract

**Background & Aims:**

*In vitro* studies suggest that hepatitis C virus (HCV) may have a direct oncogenic effect, independent of the well-established indirect mechanisms of hepatocarcinogenesis driven by chronic necroinflammation, compensatory regeneration, and fibrosis. However, evidence supporting such mechanisms in human liver tissue remains limited. This study aimed to determine whether HCV contributes directly to hepatocarcinogenesis in human liver samples.

**Methods:**

We conducted a nested case–control study within a prospective liver biopsy biobank at University Hospital Basel. Patients with chronic hepatitis C (CHC) who developed hepatocellular carcinoma (HCC) during follow-up (index cases, CHC-HCC) were identified and 1:2 propensity score-matched to CHC controls without HCC (controls, CHC-nonHCC). Matching included clinical, demographic, and virological risk factors for HCC. Liver biopsies were analyzed using RNA and whole-exome sequencing. We constructed an ensemble of linear regression models based on HCV-induced persistent transcriptomic features.

**Results:**

We identified baseline biopsies from 16 patients with CHC-HCC and 32 matched CHC-nonHCC controls. Principal component analysis of transcriptomic data significantly separated the two groups (Mann-Whitney *U* test, *p* = 0.009). Differential expression analysis revealed dysregulation of MYC target genes, the unfolded protein response, and oxidative phosphorylation in CHC-HCC samples. Notably, 615 baseline differentially expressed genes also differed from normal HCV-negative livers and remained dysregulated in non-tumour liver tissue collected years later at HCC diagnosis (*t*-test, false discovery rate <0.05). From this persistently altered gene set, we trained an ensemble of linear regression models that identified a 19-gene signature predictive of HCC risk.

**Conclusions:**

Our findings provide direct evidence from human liver biopsies that HCV contributes to hepatocarcinogenesis not only through indirect mechanisms such as inflammation and regeneration, but also through long-lasting, hepatocyte-intrinsic molecular reprogramming.

**Impact and implications:**

Hepatitis C virus has long been known to promote liver cancer through chronic inflammation and regeneration, but whether it induces durable oncogenic changes in human hepatocytes *in vivo* remained unclear. Our study demonstrates that hepatitis C virus leaves a persistent, hepatocyte-intrinsic molecular imprint detectable years before hepatocellular carcinoma diagnosis, even after viral cure. We also provide proof-of-concept for predictive transcriptomic features that can identify patients at higher risk of hepatocellular carcinoma development, which could help refine surveillance and enable targeted chemoprevention strategies.

## Highlights

• Baseline liver biopsies differ between CHC patients who subsequently develop HCC and those who do not.
• Transcriptomic differences are detectable years before HCC diagnosis.
• Many HCC-associated genes overlap with HCV-induced transcriptional changes.
• Some HCV-induced genes remain dysregulated after viral cure.
• Persistent transcriptomic features are associated with future HCC development.

## Graphical abstract

**Figure:**
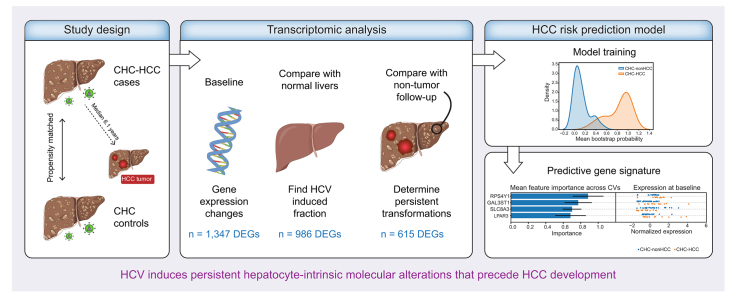

## Introduction

Hepatocellular carcinoma (HCC) largely occurs in the setting of chronic liver diseases, such as viral hepatitis, alcohol-related liver disease and metabolic dysfunction-associated steatotic liver disease (MASLD).^1^ Advanced liver fibrosis and cirrhosis are the most important risk factors in HCC carcinogenesis independent of the etiology of the underlying liver disease.^2^ Clinical features that have been associated with an increased risk of HCC are older age, race/ethnicity, male sex and portal hypertension.^2^^,^^3^ In general, hepatocarcinogenesis is conceptualised as a multistep process that involves continuous cycles of cell death and regeneration, often caused by a necro-inflammatory immune response to infected or damaged hepatocytes.^4^ Hepatocellular regeneration in the context of ongoing liver injury leads to fibrosis and the conversion of normal liver architecture into structurally abnormal nodules, ultimately resulting in cirrhosis. Ongoing cell proliferation allows for the propagation of spontaneously occurring mutations and DNA replication errors that predispose to malignant transformation of cells.^5^ As a consequence, hyperplastic nodules of regenerating hepatocytes can progress to pre-malignant dysplastic nodules that can transform into HCC.^4^

In addition to the described common mechanisms of carcinogenesis, there are likely also disease-specific oncogenic mechanisms. Genomic analysis of resected hepatitis B virus-associated HCCs has provided evidence that integration of hepatitis B virus into the human genome can activate oncogenic driver pathways.^6^^,^^7^ Similarly, the occurrence of HCC in non-cirrhotic MASLD suggests that direct carcinogenic processes, such as steatosis-induced lipotoxicity and oxidative DNA damage, may promote malignant transformation.^8^

The oncogenicity of hepatitis C virus (HCV) has been extensively studied in cell culture, where HCV has been found to induce endoplasmic reticulum and oxidative stress, interact with p53, inhibit Rb, interfere with DNA damage repair, activate β-catenin, and inhibit apoptosis.^9^ Moreover, HCV has been demonstrated to induce epigenetic and gene expression alterations that persist even after HCV cure by antiviral treatment.^10^ However, the relevance of these findings, which were obtained in cell culture models using HCV protein overexpression or infection of transformed hepatoma cells with high-multiplicity HCVcc infections, remains unclear.

In the present study, we aimed to investigate whether HCV infection can induce genomic and transcriptomic alterations associated with the development of HCC, using liver biopsy specimens from patients with chronic hepatitis C (CHC) and long-term follow-up. We conducted a nested case–control study by identifying patients from a large cohort who had undergone liver biopsy during active chronic hepatitis C and subsequently developed HCC during follow-up. Each case was propensity score–matched in a 1:2 ratio to patients with CHC who did not develop liver cancer during long-term follow-up. We identified distinct transcriptomic features in baseline biopsies that were associated with later development of HCC – providing the first direct evidence that HCV exerts oncogenic effects *in vivo*, years before cancer becomes clinically apparent.

## Patients and methods

### Patients and liver biopsies

Since 1996, patients undergoing liver biopsy for diagnostic purposes at University Hospital Basel, Switzerland, have been asked to provide written informed consent for the use of their liver tissue and blood samples in current and future research projects, including omics analyses. The study was approved by the ethics committee of the northwestern part of Switzerland (Protocol Number EKNZ 2014-099). Liver biopsies were obtained using an ultrasound-guided coaxial technique, as previously described.^11^ This collection of annotated liver biopsies now contains over 5,000 samples. This biobank was searched for patients with active CHC who developed *de novo* HCC during follow-up. Patients were 1:2 propensity-score matched with CHC controls who did not develop HCC during follow-up using the nearest neighbor method. We estimated a propensity score using logistic regression to model the probability of developing HCC based on the following variables: METAVIR fibrosis stage, age, sex, race/ethnicity, time from index biopsy to HCC diagnosis or last follow-up, Child–Pugh score, MELD score, viral load, HCV genotype, transaminase levels, HCV treatment and cure status, HIV infection, history of alcohol consumption, and diabetes mellitus. Covariate balance after propensity score matching was assessed using standardised mean differences.^12^

### RNA and whole-exome sequencing

Snap frozen biopsies were crushed in liquid nitrogen. Genomic DNA and total RNA was extracted using the ZR-Duet DNA and RNA MiniPrep Plus kit (Zymo Research) according to the manufacturer’s instructions. DNA was extracted from blood cells using the DNeasy Blood & Tissue Kit (Qiagen). We purified exomes from liver biopsies and matched blood samples using Agilent SureSelectXT HS2 V8. Matched blood samples served as controls for germline mutations. Library prep of RNA samples was done using TruSeq Stranded Total RNA Library Prep Kit with Ribo-Zero Gold (Illumina) according to the manufacturer’s specifications. RNA sequencing of baseline CHC samples was done in paired-end mode on an Illumina NovaSeq 6000 with 101 cycles. RNA sequencing (RNA-seq) of matched non-tumor follow-up samples and HCV samples used for risk score prediction was done in single-end mode on an IIllumina Hiseq 2500 with 51 or 101 cycles. RNA-seq and whole-exome sequencing (WES) were performed at the Department of Biosystems Science and Engineering ETH Zurich in Basel.

### Differential gene expression analysis

The raw reads were aligned against the NCBI human GRCh38 assembly using STAR version 2.7.9a in paired-end mode with sjdbOverhang set to 100. For comparisons with other samples in our biobank that were sequenced on Illumina machines at various cycle lengths, adapter sequences were trimmed with Trimmomatic version 0.39 with minimum length set to 41 and alignments were done in single-end mode with sjdbOverhang at 50. The read counts of each sample were summarised using FeatureCounts from the Subread package using strand information and the NCBI GRCh38 annotations. Differential gene expression was analyzed using pyDESeq2 version 0.5.3^13^ in python version 3.14.0 with sex included as a batch variable in the design formula with a significance threshold false discovery rate of 0.05. For the comparison of baseline CHC-HCC with paired non-tumor follow-up samples, paired t-tests were applied. Count normalization was done using the variance stabilizing transformation. Gene biotype and gene symbol annotation were fetched from Ensemble using biomaRt version 2.54.1. Protein coding genes with more than 10 reads in at least three samples were kept. Principal component analysis (PCA) was calculated using the top 500 most variable genes. Statistical Mann-Whitney *U* test of PC1 difference was performed with SciPy 1.6.13. Immune cell abundance was inferred from bulk RNA-seq data using the ImSig framework (R package ImSig version 1.1.3),^14^ which estimates the relative abundance of immune cell populations based on predefined, experimentally validated gene expression signatures. The analysis included signatures for neutrophils, macrophages, T cells, B cells, natural killer (NK) cells, and interferon-response programs. Full details of the signature definitions and marker gene lists are provided in the original ImSig publication. Pathway enrichment was calculated using Fisher’s exact test to assess whether differentially expressed genes (DEGs) were overrepresented in predefined gene sets compared to a background set of all detected protein-coding genes, using the Enrichr implementation in GSEApy v1.1.5^14^ ^15^ and MsigDB v2024.1.Hs.^16^^,^^17^

### Droplet digital PCR

Genomic DNA was diluted appropriately and subjected to droplet digital PCR on the Bio-Rad QX200 platform using EvaGreen reagents. Human mitochondrial DNA was quantified using specific forward (5’- TCCTAATGCTTACCGAACGA -3’) and reverse (5’- GCGTCAGCGAAGGGTTGTAG -3’) primers. Human genomic DNA was quantified with an assay targeting a single-copy gene IMAP (forward, 5’- TTTTCAGCTCCCAAGTGTCC -3’; reverse, 5’- GCCGAGAGCAGGTAGCAGT -3’). Cell numbers were estimated assuming two copies of IMAP per cell and mitochondrial DNA abundance was expressed on a per cell basis.

### Whole-exome sequencing analysis

Sequence reads were aligned to the reference human genome GRCh37 using Burrows-Wheeler Aligner (BWA, v0.7.17).^18^ Local realignment, duplicate removal and base quality adjustment were performed using the Genome Analysis Toolkit (GATK, v3.6)^19^ and Picard (http://broadinstitute.github.io/picard/, v2.25.2). The mean target coverage for the biopsies and blood samples were 47x (range 42x-55x) and 74x (range 59x-89x), respectively. Somatic single nucleotide variants and small insertions and deletions (indels) were detected using Strelka2 (v2.9.10),^20^ MuSE (v2.0.4),^21^ and CaVEMan via cgpCaVEManWrapper (v1.18.3).^22^ We filtered out single nucleotide variants and indels outside of the target regions: those with variant allelic fraction (VAF) of <1% and/or those supported by <3 reads. We excluded variants for which the tumour VAF was <5 times that of the paired non-tumour VAF. We further excluded variants identified in at least two of the blood samples in this cohort using the CreateSomaticPanelOfNormals function of MuTect2.^23^ We only considered variants detected by at least two of the three variant callers.

### Mutational statistical analysis

Statistical analyses were performed in R version 4.0.3. Correlations were assessed using the Spearman method. Time to HCC diagnosis was defined as the time interval between the time of the index biopsy and the diagnosis of HCC. Comparison of the number of somatic mutations was performed using two-sided Mann-Whitney *U* test, and *p* ≤0.05 was considered statistically significant.

### Elastic net model for predictive gene selection

Read counts were normalised by transcripts per million to account for both gene length and library size. We employed ElasticNetCV from the scikit-learn library v1.7.0^24^ to automatically tune the model's hyperparameters via 20-fold cross-validation, testing a range of l1_ratio values (0.1, 0.5, 0.7, 0.9, 0.95, 1.0) and selecting from 100 alpha values. The maximum number of iterations was set to 500. In each iteration, the expression data and group labels were resampled with replacement, and an elastic net model was fitted to the resampled dataset. Genes with non-zero coefficients in the fitted model were recorded as selected. A binary selection vector was generated per iteration, after all iterations were complete, gene selection frequency was calculated as the proportion of bootstraps in which each gene was selected. The top 100 ranked genes, with a selection frequency above 15%, were used as input for the logistic regression model.

### Logistic regression classifier with repeated stratified cross-validation

We trained a logistic regression classifier using repeated stratified K-fold cross-validation (scikit-learn; 5 folds × 100 repeats, yielding 500 models per gene subset) on the top 100 genes to further refine the selection to the most predictive features. For each value of k (top-k genes from the elastic-net ranking), models were trained using only those k features. Model performance on unseen data was evaluated using the AUC. In parallel, we recorded which genes were selected as important in each repetition. To assess the consistency of gene selection across repeated training runs, we calculated the Jaccard stability index. Within each fold, the top most influential genes were identified based on the absolute magnitude of their regression coefficients. After identifying a stable feature subset, we refit logistic regression models using a second large repeated cross-validation procedure (5 folds × 100 repeats; 500 models total). In each fold, models were trained on 80% of the data and generated out-of-fold predictions on the remaining 20%. This procedure produced an ensemble of 500 trained models with per-sample probability estimates aggregated across folds (mean ± SD), as well as fold-specific coefficient vectors enabling ensemble-level feature-importance ranking. Feature importance was quantified as the mean absolute coefficient across all 500 models, and coefficient stability was assessed using the standard deviation.

## Results

### Patients

We identified 16 patients in our liver biopsy biobank who had active CHC at the time of biopsy and subsequently developed histologically confirmed *de novo* HCC during follow-up (index cases, CHC-HCC) (Table S1). The median interval from the index biopsy to HCC diagnosis was 6.1 years (range: 0.6–12.7 years) (Fig. S1). All patients received treatment after the baseline biopsy with either direct-acting antivirals or interferon-based therapies and were cured from HCV. At the time of HCC diagnosis, 12 patients were HCV negative. One patient, due to noncompliance, had persistent HCV infection despite two treatment courses. Another patient was undergoing antiviral therapy at the time of HCC diagnosis, while three patients were treated after HCC diagnosis. Patients with CHC-HCC were propensity-score matched 1:2 with patients with CHC who did not develop HCC during follow-up (controls, CHC-nonHCC). The median follow-up of these patients was 10.2 years (range 2.6–19.4 years) (Fig. S1). All patients with CHC-nonHCC had active chronic HCV infection at the time of biopsy and received antiviral therapy thereafter; all except one patient, who relapsed following interferon-based treatment, achieved sustained virological cure of HCV. Matching was based on established HCC risk factors, as detailed in the methods section. There were no clinically relevant differences in the prevalence of these risk factors between the CHC-HCC and CHC-nonHCC groups (Table 1).

**Table 1:** Patient characteristics.

	Total	Controls	HCC cases
	N = 48	n = 32	n = 16
Age at time of index biopsy, years	52.5 (47.0-60.5)	50.5 (46.5-59.5)	57.0 (48.5-65.5)
Sex			
Female	15 (31%)	11 (34%)	4 (25%)
Male	33 (69%)	21 (66%)	12 (75%)
Race			
Asian	2 (4%)	0 (0%)	2 (12%)
Black	2 (4%)	1 (3%)	1 (6%)
Caucasian	44 (92%)	31 (97%)	13 (81%)
HCV as underlying liver disease	48 (100%)	32 (100%)	16 (100%)
Histological signs of ALD or MASLD	30 (62%)	19 (59%)	11 (69%)
METAVIR grading			
1	1 (2%)	1 (3%)	0 (0%)
2	20 (42%)	14 (44%)	6 (38%)
3	27 (56%)	17 (53%)	10 (62%)
METAVIR staging			
3	7 (15%)	6 (19%)	1 (6%)
4	41 (85%)	26 (81%)	15 (94%)
Time from index biopsy to diagnosis of HCC or follow-up (days)	3,155.0 (2,035.5-4,456.0)	3,876.5 (2,659.5-4,823.0)	2,035.5 (1,127.5-3,242.5)
Child-Pugh score	5.4 (0.7)	5.4 (0.7)	5.3 (0.8)
MELD score	6.9 (1.6)	6.9 (1.8)	7.0 (1.0)
HCV genotype			
1	29 (60%)	19 (59%)	10 (62%)
2	4 (8%)	3 (9%)	1 (6%)
3	13 (27%)	9 (28%)	4 (25%)
4	2 (4%)	1 (3%)	1 (6%)
HCV viral load, IU/ml	1.2e^+06^ (323,275.5-2.1e^+06^)	1.2e^+06^ (276,791.0-1.8e^+06^)	1.5e^+06^ (530,000.0-2.9e^+06^)
Treatment			
IFN-based	18 (38%)	14 (45%)	4 (25%)
DAA	29 (62%)	17 (55%)	12 (75%)
SVR	46 (96%)	31 (97%)	15 (94%)
HIV co-infection	6 (12%)	4 (12%)	2 (12%)
ALT, IU/L	102.0 (54.5-156.0)	97.5 (50.5-144.0)	124.0 (56.5-177.5)
AST, IU/L	85.5 (53.5-127.0)	66.0 (50.0-114.0)	108.5 (88.5-147.0)
Smoking status	18 (38%)	13 (41%)	5 (31%)
Diabetes mellitus	7 (15%)	3 (9%)	4 (25%)
Metformin	6 (12%)	2 (6%)	4 (25%)
Body mass index, kg/m^2^	25.6 (5.3)	24.8 (4.5)	28.4 (7.6)

ALD, alcohol-related liver disease; ALT, alanine aminotransferase; AST, aspartate aminotransferase; DAA, direct-acting antiviral; HCC, hepatocellular carcinoma; HCV, hepatitis C virus; IFN, interferon; MASLD, metabolic dysfunction-associated steatotic liver disease; MELD, model for end-stage liver disease.

Data are presented as mean (SD) or median (IQR) for continuous and n (%) for categorical measures, respectively.

### Transcriptome analysis identifies differentially expressed genes and dysregulated pathways associated with HCC development

**Fig. 1:**
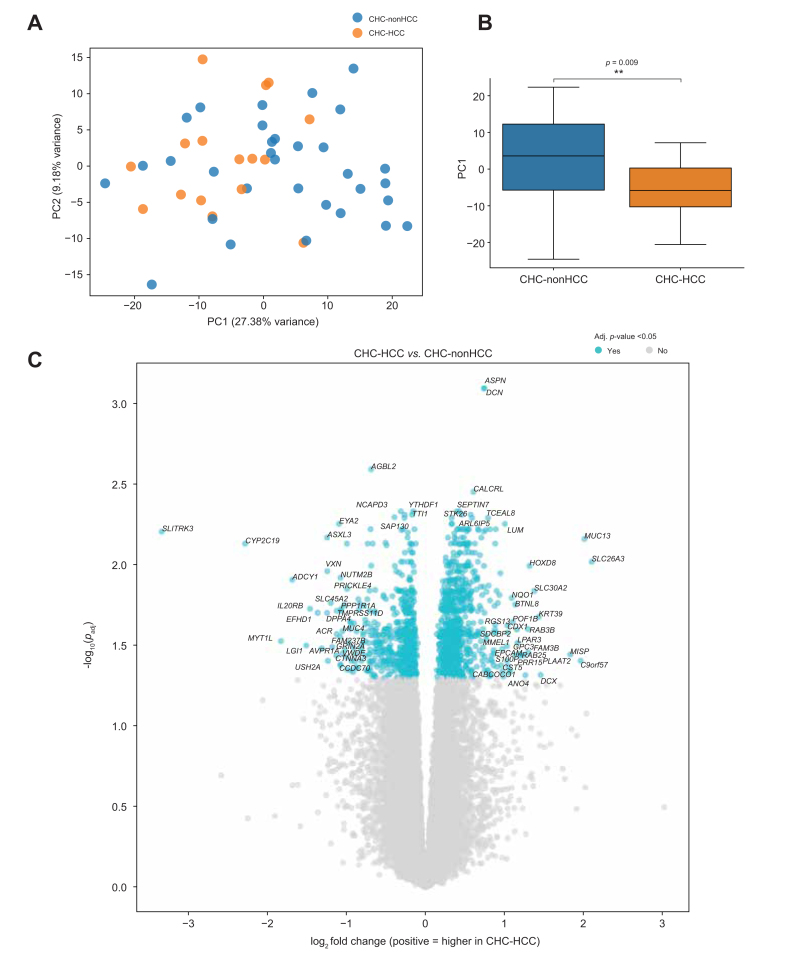
Principal component analysis and differential gene expression in CHC patients. (A) Principal component analysis of liver biopsy transcriptomes, based on the 500 most variable genes. Each point represents an individual patient at baseline. (B) Boxplots showing the distribution of the two patient groups along PC1. A Mann–Whitney *U* test reveals a significant difference between groups (*p* = 0.009). (C) Volcano plot illustrating differential gene expression between CHC-nonHCC and CHC-HCC samples. Each dot represents a gene, plotted by log_2_ fold change (x-axis) and –log_10_ (adjusted *p* value) (y-axis). CTAG2 was filtered out for visualization purposes (*p* value = 0.94 log_2_ fold change = 33). Genes with an absolute log_2_ fold change above 1 or adjusted *p* value below 0.005 were annotated on the plot. Positive values indicate higher expression in CHC-HCC. Genes significantly differentially expressed (FDR <0.05) are highlighted in light blue. CHC, chronic hepatitis C; FDR, false discovery rate; HCC, hepatocellular carcinoma; PC, principal component.

An unbiased PCA of the 500 most variably expressed genes found in the RNA-seq data of the 16 CHC-HCC and 32 CHC-nonHCC samples revealed a significant contribution of sex to gene expression variation between samples but no clear separation of CHC-HCC from CHC-nonHCC samples (data not shown). Because most of these sex-associated transcriptional differences are well described and not directly related to HCC risk, we adjusted for sex to better resolve gene expression changes associated with future HCC development. After correction for sex, PCA revealed a separation tendency of CHC-HCC from CHC-nonHCC samples (Fig. 1A) with a significant difference between groups in principal component 1 (Mann-Whitney *U* test, *p* = 0.009) (Fig. 1B).

Differential gene expression analysis identified 1,347 DEGs with a Benjamini-Hochberg adjusted *p* value (*p*adj) <0.05 (Fig. 1C, Table S2). One of the most strongly downregulated genes in CHC-HCC was *CYP2C19* (log_2_FC: –2.28, *p*_adj_ = 0.007), a finding consistent with our previous report of *CYP2C19* downregulation in non-tumor liver tissue from patients with HCC.^25^ Among the 56 genes with more than two-fold differential expression, 24 have previously been linked to HCC in the literature (Table S3), supporting the biological relevance of the transcriptional changes observed in the CHC-HCC group.

**Fig. 2:**
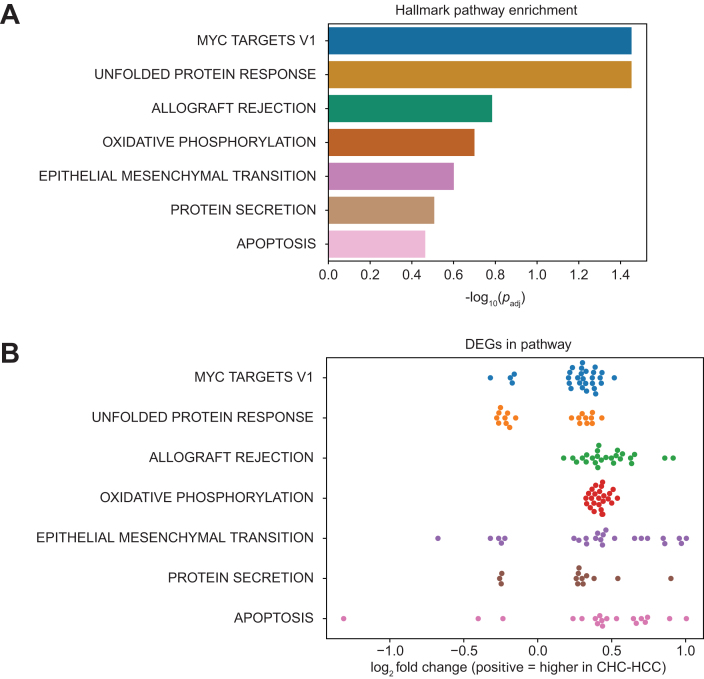
Hallmark pathway enrichment analysis of DEGs between patients with CHC-nonHCC and CHC-HCC. (A) Horizontal bar plot showing Hallmark pathways significantly enriched among DEGs from liver biopsy transcriptomics, identified using Fisher’s exact test. The x-axis shows –log_10_ (adjusted *p* value). (B) Swarm plot displaying the log_2_ fold change of individual DEGs within each enriched Hallmark pathway. Each point represents a single gene, grouped by pathway. CHC, chronic hepatitis C; DEGs, differentially expressed genes; HCC, hepatocellular carcinoma.

Hallmark pathway enrichment analysis of the DEGs identified several pathways significantly enriched in CHC-HCC. Among them, MYC Targets V1 was the most significantly enriched (*p* = 0.001, *p*_adj_ = 0.035; 29 out of 200 genes) (Fig. 2A). To assess whether the MYC-driven transcriptional programme translated into detectable protein-level changes, we also examined c-MYC expression and hepatocyte proliferation by Ki-67 immunohistochemistry in baseline FFPE biopsies; however, no significant differences were observed between patients with CHC-HCC and CHC-nonHCC (Fig. S2). Another significantly enriched pathway was the unfolded protein response (*p* = 0.001, *p*_adj_ = 0.035; 19 out of 114 genes), with roughly half the genes upregulated and the other half downregulated in CHC-HCC samples (Fig. 2B).

The allograft rejection pathway was also enriched in CHC-HCC (*p* = 0.001, *p*_adj_ = 0.164; 25 out of 192 genes), with all associated genes showing higher expression in CHC-HCC samples (Fig. 2B). Since these genes are predominantly expressed in immune cells, we considered whether differences in immune cell infiltration might underlie this signal. However, analysis of RNA-seq data revealed no significant differences in immune cell signatures between CHC-HCC and CHC-nonHCC samples, although both groups showed elevated immune signatures compared to normal liver (Fig. S3).

Lastly, the oxidative phosphorylation (OxPhos) pathway was enriched in CHC-HCC (*p* = 0.016, *p*_adj_ = 0.200; 25 out of 200 genes) (Fig. 2A), with all OxPhos genes upregulated in this group (Fig. 2B). Of note, all differentially expressed OxPhos genes were nuclear-encoded, and mitochondrial gene expression was comparable between groups (Fig. S4A), indicating that this transcriptional activation is not due to increased mitochondrial mass. This was further supported by droplet digital PCR, which showed no difference in mitochondrial DNA content per cell (Fig. S4B).

### Genomic analysis

HCC carcinogenesis is underpinned by the accumulation of somatic genetic alterations. One could hypothesise that the number of somatic mutations would be higher in CHC-HCC than CHC-nonHCC. Through WES of the subset of 13 patients with CHC-HCC and 25 with CHC-nonHCC from whom matched blood samples were available, we identified a median of 9 (range 3-59) and 4 (range 1-33) somatic mutations, respectively (Fig. S5A; Table S4). Moreover, among CHC-HCC, we found a strong negative correlation between somatic mutation burden and time to HCC diagnosis, indicating that higher mutation burdens were associated with shorter times to HCC diagnosis (R = -0.82, Fig. S5B). Of note, WES was used here to quantify the global somatic mutation burden rather than to identify specific oncogenic driver mutations, as the baseline biopsies were taken years before HCC diagnosis and likely from sites unrelated to the future tumour. Accordingly, no mutations were identified in HCC mutational driver genes, and we did not observe any recurrent mutations or mutated genes that were overrepresented in the CHC-HCC group (Table S4).

### A subset of 615 baseline CHC-HCC DEGs is HCV-induced and remains dysregulated in non-tumor liver tissue at the time of HCC diagnosis

**Fig. 3:**
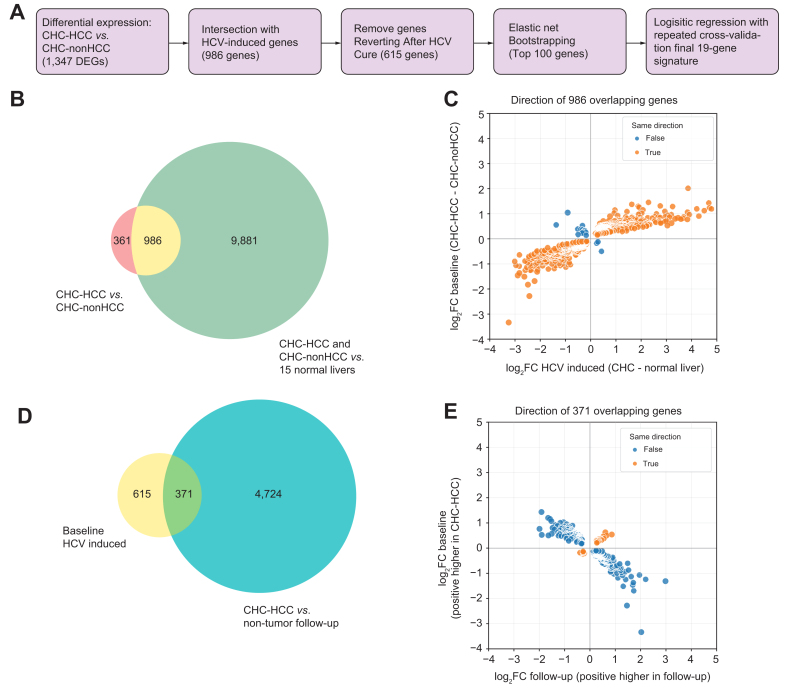
Persistent transcriptomic dysregulation after HCV cure. (A) Flowchart of our strategy aimed to identify a predictive signature of HCC development based on HCV-induced persistent transcriptional changes. (B) Venn diagram. Circles indicate the sets of genes that are significantly differentially expressed (DEGs; FDR ≤0.05). Red: baseline CHC-nonHCC *vs*. CHC-HCC. Green: CHC baseline biopsies with 15 normal livers with no HCV infection or other liver disease. Overlaps denote shared DEGs; the orange intersection highlights 986 genes that are different at baseline and different compared to normal livers without an active HCV infection. (C) Scatter plot. Out of the 986 overlapping genes in (B) the fold-change in each comparison is displayed on each axis, CHC *vs*. normal livers on the x-axis and baseline CHC-nonHCC *vs.* CHC-HCC on the y-axis. Genes with opposite directionality (enriched in CHC and depleted in CHC-HCC or depleted in CHC and enriched in CHC-HCC) are colored in blue. (D) Venn diagram. Circles indicate the sets of genes that are significantly differentially expressed (DEGs; FDR ≤0.05). Yellow: overlap from (B). Blue: Paired comparison of CHC-HCC baseline biopsies with their matched non-tumor follow-up biopsies taken at time of HCC diagnosis. Overlaps denote shared DEGs; the orange intersection highlights 371 genes that are different at baseline and different compared to follow-up non-tumor biopsies. (E) Scatter plot. Out of the 371 overlapping genes in (D) the fold-change in each comparison is displayed on each axis, baseline CHC-nonHCC *vs*. CHC-HCC on the y-axis and follow-up CHC-HCC *vs.* follow-up on the x-axis. Genes with opposite directionality (enriched in CHC-HCC and depleted in follow-up or depleted in CHC-HCC and enriched in follow-up) are colored in blue. CHC, chronic hepatitis C; DEGs, differentially expressed genes; FDR, false discovery rate; HCC, hepatocellular carcinoma; HCV, hepatitis C virus.

Our strategy aimed to identify a predictive signature of HCC development based on HCV-induced persistent transcriptional changes, by first defining genes associated with HCC risk within patients with CHC, then restricting these to HCV-related genes that remain altered after viral cure (Fig. 3A). The baseline biopsies used in this study were obtained from patients with active CHC, most of whom were later cured of the infection. We compared these CHC biopsies with 15 normal HCV-negative livers (Table S5, also described in^26^) to identify DEGs associated with chronic HCV infection. This comparison revealed 10,867 genes significantly altered by chronic infection, of which 986 overlapped with the 1,347 DEGs identified at baseline between patients who developed HCC (CHC-HCC) and those who did not (CHC-nonHCC) (Fig. 3B). We further examined the directionality of gene expression changes within this overlapping gene set. Most overlapping genes exhibited concordant directionality between the HCV-induced comparison (CHC *vs*. normal liver) and the baseline CHC-HCC *vs*. CHC-nonHCC comparison (Fig. 3C). Although directionality was largely consistent across comparisons, fold-change magnitudes were generally smaller in the CHC-HCC *vs.* CHC-nonHCC comparison than in the HCV-infected *vs*. normal liver comparison.

At the time of HCC diagnosis in the CHC-HCC group, matched tumor and non-tumor liver biopsies were collected. A paired t-test comparing these non-tumor follow-up biopsies with the corresponding baseline CHC-HCC samples identified 5,095 DEGs (Fig. 3D). As expected, Hallmark pathway enrichment analysis of these DEGs revealed a strong interferon response signature in the baseline biopsies, which resolved following HCV cure (Fig. S6A). The most differentially expressed genes identified in the Interferon pathways were CXCL10, ISG15 and OASL which all showed more than 6-fold enrichment in the baseline CHC-HCC samples, while CRP, part of the xenobiotic metabolism pathway, was more than 8-fold enriched in follow-up samples (Fig. S6B). The vast majority of DEGs identified in comparison with follow-up samples overlapped with HCV-induced genes (Fig. S6C). We compared the 986 baseline HCV-induced genes with the 5,095 DEGs identified at follow-up and found 371 overlapping DEGs (Fig. 3D). These genes represent changes at baseline that may have reverted after HCV cure and would not constitute persistent transformations (Fig. 3E). We excluded these 371 genes, which left us with a selection of 615 HCV-induced persistent DEGs that may represent stable, HCC risk-associated changes.

### Predictive models

**Fig. 4:**
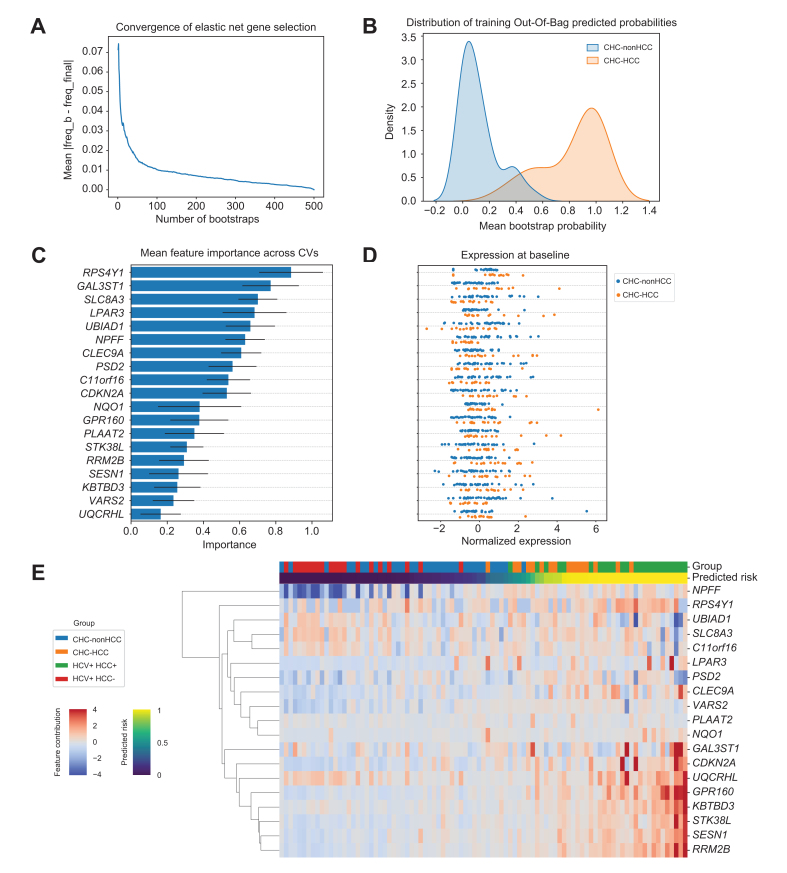
Feature selection and training of linear regression models for HCC risk prediction. (A) Convergence of gene selection frequencies during elastic net bootstrapping. Line plot showing the L1 distance (sum of absolute differences between current selection *vs*. final selection) between the gene selection frequency vectors of consecutive iterations across 500 bootstrap iterations. The x-axis represents the iteration number, and the y-axis shows the L1 distance. A decreasing L1 distance indicates stabilization of gene selection, reflecting model convergence. (B) Histogram of predicted probabilities across all baseline samples using the top 19 selected genes in 500 linear regression models with repeated resampling. In each iteration, the samples were split into training/test sets, and predicted probabilities were recorded for test samples in each iteration. A low probability denotes low risk of HCC and high probability a high risk. (C) Feature importance of the selected top 19 genes across all 500 linear regression models. (D) Expression abundances of the selected top 19 genes at baseline. Expression values were normalised by TPM and scaled using scikit-learn standard scaler. (E) The trained logistic regression models were applied to two external liver biopsy cohorts: 20 HCV+ HCC− biopsies from patients without fibrosis and no HCC during follow-up (low-risk),^28^ and 23 non-tumour biopsies collected at HCC diagnosis in patients with active CHC (high-risk).^26^ Predicted risk for each sample was calculated as the mean probability across 500 cross-validated models. The heatmap displays per-feature contributions to the predicted risk, where positive values indicate an increase and negative values a decrease in predicted risk. Samples are arranged by hierarchical clustering based on feature contributions. CHC, chronic hepatitis C; HCC, hepatocellular carcinoma; HCV, hepatitis C virus; TPM, transcripts per million.

Next, we tested whether the 615 persistently dysregulated genes could be used to develop a predictive model for HCC risk in patients with CHC. Genes were ranked by their selection frequency in an elastic net regression model, which combines L1 and L2 regularization to handle correlated features.^27^ Robustness was ensured by 500 bootstrap iterations with resampling. In each resample, the model was trained on a stratified bootstrap sample, and the presence or absence of each gene in the final regularised coefficient vector was recorded. Genes were ranked by their selection frequency across bootstraps. We observed convergence of gene selection frequencies after ∼100 iterations, as indicated by stabilisation of the L1 distance (Fig. 4A). Although the elastic net model converged toward a stable subset of consistently selected genes, the distribution of out-of-bag R^2^ values did not stabilise, indicating that performance estimates based on the bootstrap procedure were unreliable for this dataset (Fig. S7A). The elastic-net step was therefore used strictly for dimensionality reduction and ranking of genes, not for final model estimation or performance inference (Fig. S7B). Several genes – *UBIAD1, RPS4Y1, LPAR3, GAL3ST1* and *PSD2* – were selected in over 70% of iterations, indicating a strong and consistent predictive signal. Although the expression differences of individual genes were small, we hypothesised that the combined features may be strong enough to train a predictive model. Based on the elastic-net ranking, 100 genes with a selection frequency exceeding 15% were selected as candidate predictive features for subsequent analyses.

Next, we trained a logistic regression classifier using Repeated Stratified KFold (scikit-learn) cross-validation (5 folds × 100 repeats; 500 models per gene subset) using these 100 genes to further reduce the selection to the most predictive genes. For each value of k (top-k genes from the elastic-net ranking), models were trained using only those k features. We recorded both how well the model predicted unseen samples and which genes were selected as important in each repetition. We found that a set of 20 genes gave the best model accuracy and stability as seen by the high mean AUC and high Jaccard stability across repetitions (Fig. S7C and D). However, model calibration improved upon exclusion of CHIC2, resulting in a final 19-gene predictive signature (Fig. S7E).

This final group of 19 reliable genes was used to train a large ensemble of 500 simple linear regression prediction models. Each model was trained on part of the data and tested on the remaining samples with excellent sensitivity and specificity (Figs 4B and S7F). The ensemble enables robust estimates of each sample’s predicted probability (based on many models rather than a single one) with a measure of uncertainty for each prediction and a ranking of how strongly each gene contributed to the prediction across all models (Fig. 4C). We found that *RPS4Y1, GAL3ST1* and *SLC8A3* consistently ranked as the most important features across models, despite exhibiting only modest expression differences between groups (Fig. 4D).

Although a fully independent, well-matched validation cohort is not available, we performed a qualitative “sanity check” to assess the model’s ability to generalise beyond the training data by applying it to two external biopsy cohorts: 20 HCV+ HCC− biopsies from patients without fibrosis and no HCC during follow-up (low-risk),^28^ and 23 non-tumour biopsies collected at HCC diagnosis in patients with active CHC (high-risk).^26^ These datasets were not used during gene selection or model training. They were not intended as formal validation cohorts, but rather to provide an independent qualitative assessment of whether the identified transcriptional signal can be observed in biologically distinct patient groups. Accordingly, normalization was performed independently for each dataset, and no cross-cohort batch correction was applied. Predicted risk was calculated as the mean probability across 500 cross-validated models. In parallel, we computed per-feature contributions to the prediction, where positive values indicate an increase in predicted risk and negative values a decrease.

These data were visualised jointly with the training samples using a heatmap of feature contributions with hierarchical clustering, alongside annotations for sample group and predicted risk, enabling comparison of model-derived patterns across training and external cohorts (Fig. 4E). These findings suggest that our models based on 19-gene expression signatures may serve as a useful molecular tool for early HCC risk stratification in patients with CHC.

## Discussion

Advanced fibrosis and cirrhosis remain the dominant clinical predictors of HCC, yet cell culture studies have long suggested that HCV can directly activate oncogenic pathways within hepatocytes.^2^^,^^9^^,^^29^ Despite the rather small sample size, careful matching using a large liver biopsy biobank enabled us to identify a robust set of differentially expressed genes – many previously linked to hepatocarcinogenesis – that distinguished future HCC cases from controls. These findings suggest that HCV leaves a lasting molecular imprint that may contribute to HCC risk, even after viral clearance. Importantly, the term “HCV-induced” in this study refers to gene expression changes observed in the context of chronic HCV infection and does not imply specificity to HCV, as similar biological pathways may also be activated in other chronic liver diseases.

Necro-inflammatory injury is a well-established driver of hepatocarcinogenesis, including in CHC.^4^ In our study, propensity matching on Metavir activity grade ensured that patients with CHC-HCC and CHC-nonHCC had comparable levels of histological inflammation at baseline. To further assess potential differences in immune-mediated mechanisms, we deconvoluted bulk RNA-seq data using ImSig (Fig. S2) and found that the relative abundance of key immune cell populations – including neutrophils, macrophages, T, B, and NK cells – as well as interferon-response signatures, was highly similar between groups. This allowed us to identify hepatocyte-intrinsic transcriptional programs, most likely reflecting stable epigenetic imprints of HCV infection, that are associated with future HCC development.

The most significantly enriched pathway in CHC-HCC compared to CHC-nonHCC was MYC Targets V1, reflecting coordinated upregulation of genes regulated by the MYC oncogene. MYC is a central transcription factor in cell cycle progression, proliferation, and metabolism, and plays a pivotal role in many human cancers.^30^ In experimental models, MYC overexpression alone is sufficient to initiate liver tumorigenesis,^31^^,^^32^ and it is frequently activated in human HCC through genomic amplification or upstream pathway dysregulation.^33^ The presence of a MYC-driven transcriptional programme years before HCC diagnosis suggests that HCV infection may induce a proliferative state in hepatocytes that persists beyond viral clearance.

Another pathway significantly dysregulated in CHC-HCC was the unfolded protein response (UPR), a cellular stress response triggered by the accumulation of misfolded proteins in the endoplasmic reticulum. While transient UPR activation helps restore proteostasis, chronic or dysregulated UPR signalling is increasingly recognised as a contributor to carcinogenesis.^34^ HCV infection has been shown to activate the UPR in Huh7 hepatoma cells, highlighting a direct link between viral replication and endoplasmic reticulum stress.^35^ In diethylnitrosamine-induced liver cancer models, UPR components have been implicated in both tumour initiation and progression.^36^ UPR activation has also been documented in human HCC tissues.^37^^,^^38^ The presence of a UPR gene expression signature at baseline in CHC-HCC samples may reflect early and persistent cellular stress responses that contribute to a tissue environment more permissive to malignant transformation.

OxPhos was another pathway upregulated in CHC-HCC. This may at first seem counterintuitive, as many cancers favour glycolysis (“Warburg effect”) over OxPhos. However, in a premalignant liver, enhanced OxPhos might be an adaptive response to the increased energy demand of proliferating cells. Indeed, we have previously found enhanced OxPhos in an mTOR-driven mouse model of HCC.^39^ Mechanistically, elevated OxPhos activity may contribute to carcinogenesis by increasing the production of reactive oxygen species, which promote genomic instability and DNA damage – key drivers of malignant transformation.

Indeed, the median number of somatic mutations was higher in the CHC-HCC group, although the difference was not statistically significant, likely owing to the limited sample size. However, the strong inverse correlation between mutation burden and time to HCC diagnosis supports a role for increased mutagenesis in driving cancer risk. Notably, we did not observe enrichment of known HCC driver mutations. This is not unexpected, given that the baseline biopsies represent random liver sampling, and the subsequent HCCs likely arose in different hepatic regions years later. Rather than capturing early neoplastic lesions, our findings suggest the presence of a broader pro-tumorigenic “field effect” in a subset of HCV-infected patients predisposed to cancer development. The identification of OxPhos as one of the most deregulated pathways in future HCC cases provides a rationale for targeting oxidative stress with antioxidants such as glutathione or its precursors. Such chemo-preventive strategies may help counteract reactive oxygen species-driven mutagenesis in at-risk patients following HCV cure.

Among the 1,347 genes that distinguished CHC-HCC from CHC-nonHCC at baseline, 615 were induced by HCV infection and remained dysregulated in paired, non-tumour liver biopsies collected years later at the time of cancer diagnosis, indicating a persistent, HCC-associated transcriptional programme. Using these 615 “scar” genes, we derived a 19-gene signature through an ensemble of 500 linear regression models that consistently discriminated future HCC cases from matched controls in our study cohort. The signature showed good discrimination when applied to two independent HCV biopsy datasets representing low-risk and high-risk patient groups, respectively. Derived from a carefully matched cohort and based on genes that remain persistently dysregulated years before HCC diagnosis, this ensemble-based signature has strong potential as a quantitative molecular tool for identifying patients with CHC who remain at elevated risk for HCC despite virologic cure. Any such assay, however, will need to add value beyond established clinical predictors – age, sex, fibrosis stage, steatotic liver disease, diabetes, and other routine laboratory-based scores that already achieve respectable discrimination.^40^ Prospective studies that integrate our 19-gene panel with these clinical variables will be essential to determine whether transcriptomic profiling meaningfully refines risk stratification or identifies a subset of patients who would benefit from intensified surveillance. A practical challenge for implementing this approach is that few patients with cured HCV routinely undergo liver biopsy, and our analyses were performed on fresh-frozen tissue rather than routinely available FFPE samples. Nevertheless, it may be worthwhile to consider a new biopsy in selected patients if the signature is validated in prospective trials and if chemopreventive therapies can be offered to those identified as high risk. Beyond its potential clinical relevance, our study demonstrates that HCV can contribute to hepatocarcinogenesis not only through chronic inflammation and regeneration, but also by inducing persistent, hepatocyte-intrinsic molecular changes.

## Abbreviations

CHC, chronic hepatitis C; DEG, differentially expressed gene; HCC, hepatocellular carcinoma; HCV, hepatitis C virus; MASLD, metabolic dysfunction-associated steatotic liver disease; OxPhos, oxidative phosphorylation; PCA, principal component analysis; SNV, single nucleotide variants; UPR, unfolded protein response; VAF, variant allele frequency.

## Authors’ contributions

Tuyana Boldanova: Conceptualization, Investigation, Visualization, Writing; Fredrik Trulsson: Formal analysis, Data Curation, Visualization, Writing; Fahim Ebrahimi: Formal analysis, Writing; Andrej Benjak: Formal analysis; Matthias S. Matter: Resources; Aleksei Suslov: Investigation, Visualization; Stefan Wieland: Conceptualization; Charlotte K. Y. Ng: Formal analysis, Data Curation, Visualization, Writing; Markus H. Heim: Conceptualization, Writing, Supervision, Funding acquisition.

## Data availability

Sequencing data are available at the European Genome-phenome Archive under accessions EGAS50000001096 (whole-exome sequencing), EGAS50000001097 (RNA-seq: Baseline CHC) and EGAS50000001098 (RNA-seq: Matched non-tumor follow-ups and HCV biopsies).

## Code availability

All code used in the study have been deposited at Github https://github.com/Trulsson/Transcriptomic_profiles_in_CHC_predict_HCC.

## Declaration of generative AI and AI-assisted technologies in the writing process

During the preparation of this work the author(s) used ChatGPT 5.2 in order to check grammar and readability. After using this tool/service, the author(s) reviewed and edited the content as needed and take full responsibility for the content of the publication.

## Financial support

The project was supported by the ERC Synergy Grant 609883 and the Swiss Cancer Research project grant KFS-5486-02-2022.

## Conflict of interest

The authors declare no conflicts of interest related to this work.

Please refer to the accompanying ICMJE disclosure forms for further details.

## Supplementary data

Multimedia compoent 1

Multimedia compoent 2

Multimedia compoent 3

Multimedia compoent 4

Multimedia compoent 5

Multimedia compoent 6
