# Supplementary material for: Hepatitis C virus can induce gene expression changes associated with hepatocarcinogenesis
Source: JHEP Rep. 2026 May 29;8(8):101897. doi: 10.1016/j.jhepr.2026.101897 (PMC13380720; doi:10.1016/j.jhepr.2026.101897)
Supplement: Multimedia compoent 1 [file mmc1.pdf]

Hepatitis C virus can induce gene expression changes associated with hepatocarcinogenesis

Tuyana Boldanova, Fredrik Trulsson, Fahim Ebrahimi, Andrej Benjak, Matthias S. Matter, Aleksei Suslov, Stefan Wieland, Charlotte K. Y. Ng, Markus H. Heim

Table of contents

Fig. S1……………………………………………………………………………………..2

Fig. S2……………………………………………………………………………………..3

Fig. S3……………………………………………………………………………………..4

Fig. S4……………………………………………………………………………………..6

Fig. S5……………………………………………………………………………………..8

Fig. S6……………………………………………………………………………………..9

Fig. S7…………………………………………………………………………………11

Table S1……………………………………………………….………separate excel file

Table S2……………………………………………………….………separate excel file

Table S3……………………………………………………….…………………………...13

Table S4……………………………………………………….………separate excel file

Table S5……………………………………………………….…………………………...15

Supplementary references…………………………………………………………16

Supp. Figure 1

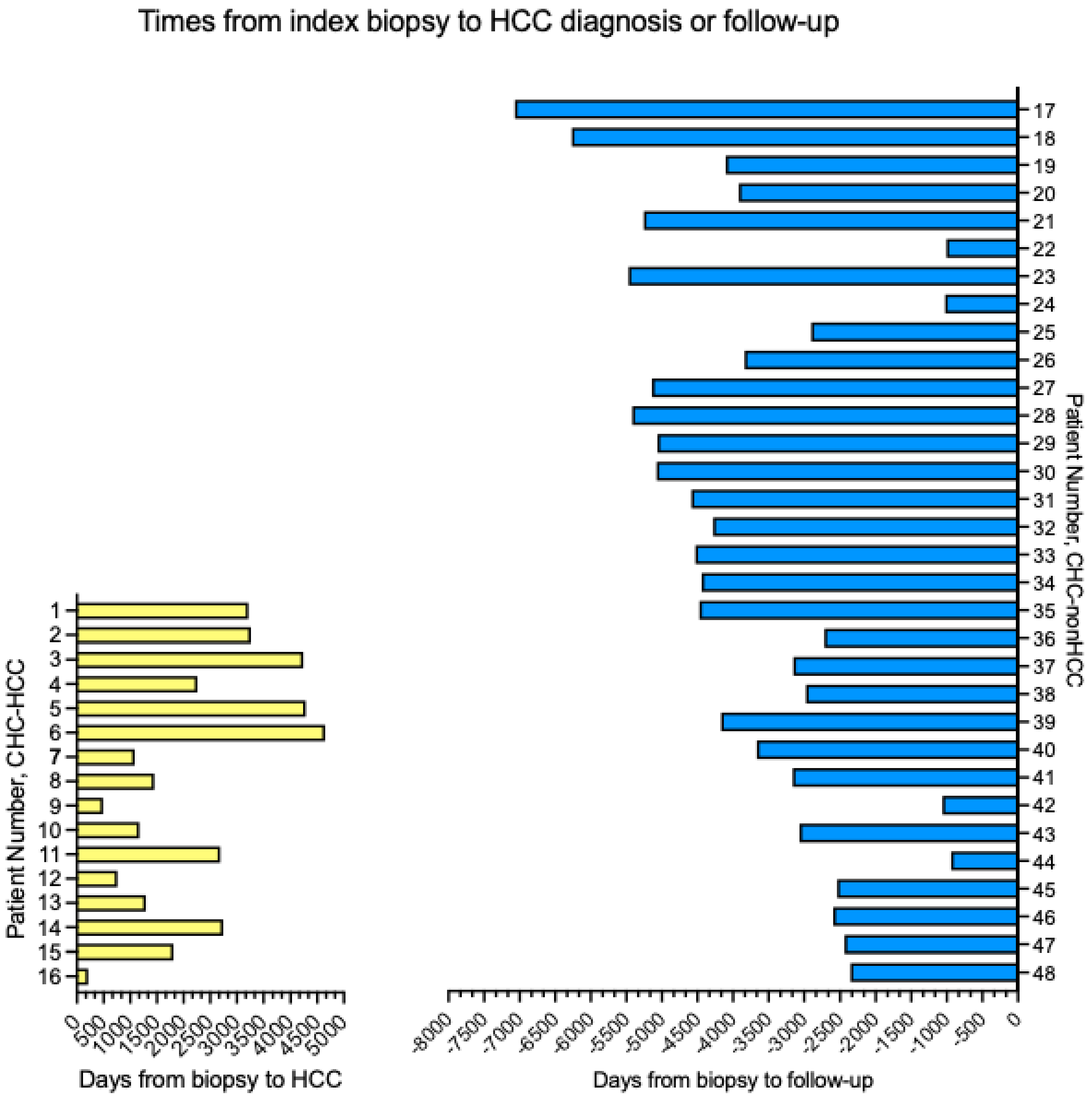

Fig. S1. Time from index liver biopsy to clinical outcomes in CHC patients. Horizontal bar graphs showing the duration (in days) from the index liver biopsy to clinical follow-up or hepatocellular carcinoma (HCC) diagnosis in two patient groups.

Left panel: Time to HCC diagnosis in patients who developed HCC (*CHC-HCC* group).

Right panel: Time to last clinical follow-up in patients who did not develop HCC (*CHC-nonHCC* group). Each bar represents an individual patient.

Supp. Figure 2

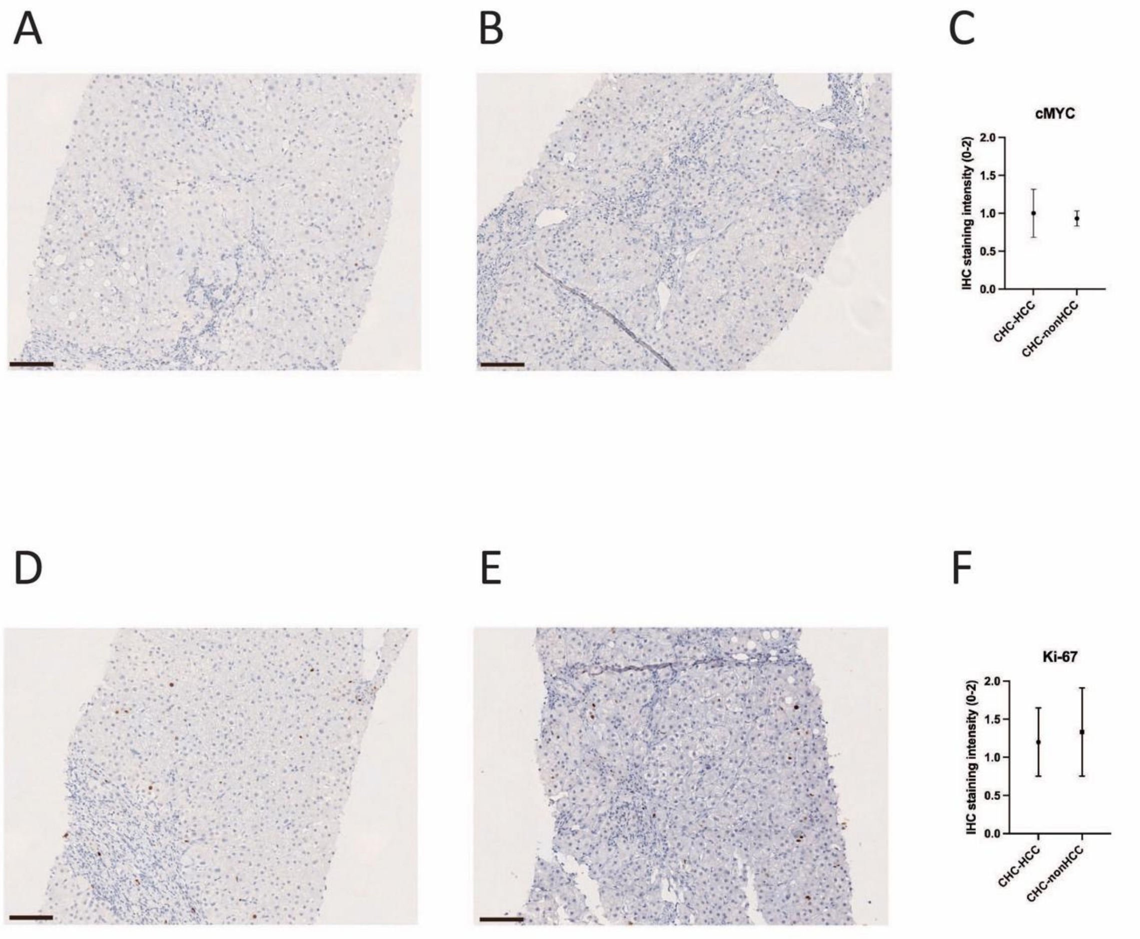

Fig. S2. Immunohistochemical staining for cMYC and Ki-67.

A: Representative cMYC IHC from CHC-HCC patient.

B: Representative cMYC IHC from CHC-nonHCC patient.

C: Unpaired t test with Welch's correction between CHC-HCC and CHC-nonHCC groups for cMYC IHC, difference not significant.

D: Representative Ki-67 IHC from CHC-HCC patient.

E: Representative Ki-67 IHC from CHC-nonHCC patient.

F: Unpaired t test with Welch's correction between CHC-HCC and CHC-nonHCC groups for Ki-67 IHC, difference not significant.

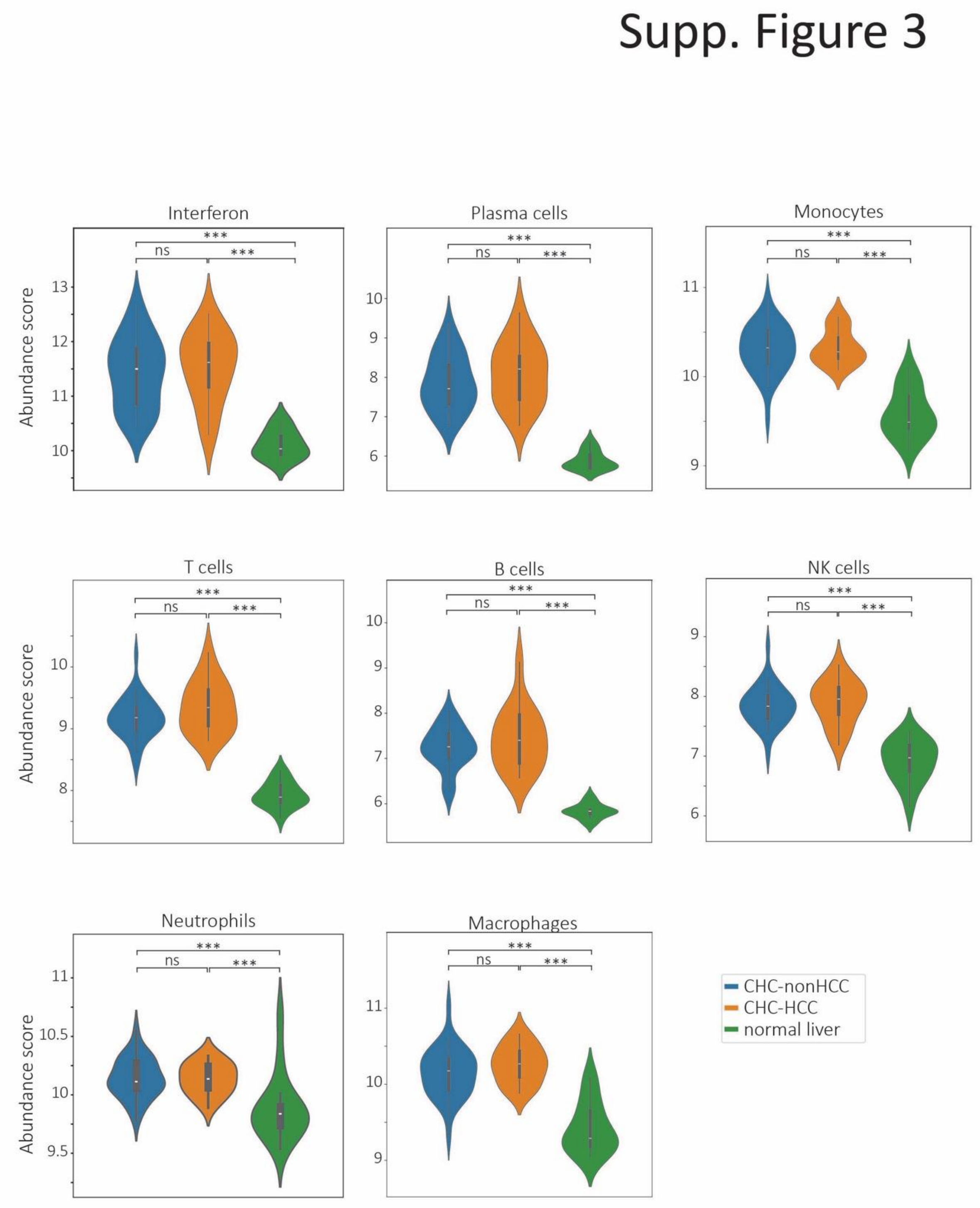

Fig. S3. Inferred immune cell abundance and interferon response across CHC and control liver biopsies.

Violin plots showing the inferred relative abundance of immune cell types and interferon response signatures across three groups: *CHC-HCC*, *CHC-nonHCC* and normal liver tissue. Normal liver tissue controls were derived from RNA-Seq data of 15 liver biopsies from individuals without liver disease (Supplementary Table 5), with normal histology and liver

function tests, as previously described [1]. Immune cell abundance was estimated from bulk transcriptomic data using the ImSig R package. Each violin represents the distribution of inferred abundance for a given immune cell type within each group. Pairwise group comparisons were performed using the Wilcoxon rank-sum test. P-values were adjusted for multiple testing across all immune cell types and comparisons using the Benjamini–Hochberg method. Statistical significance is indicated as follows: ns = not significant; * FDR < 0.05; ** FDR < 0.01; *** FDR < 0.001.

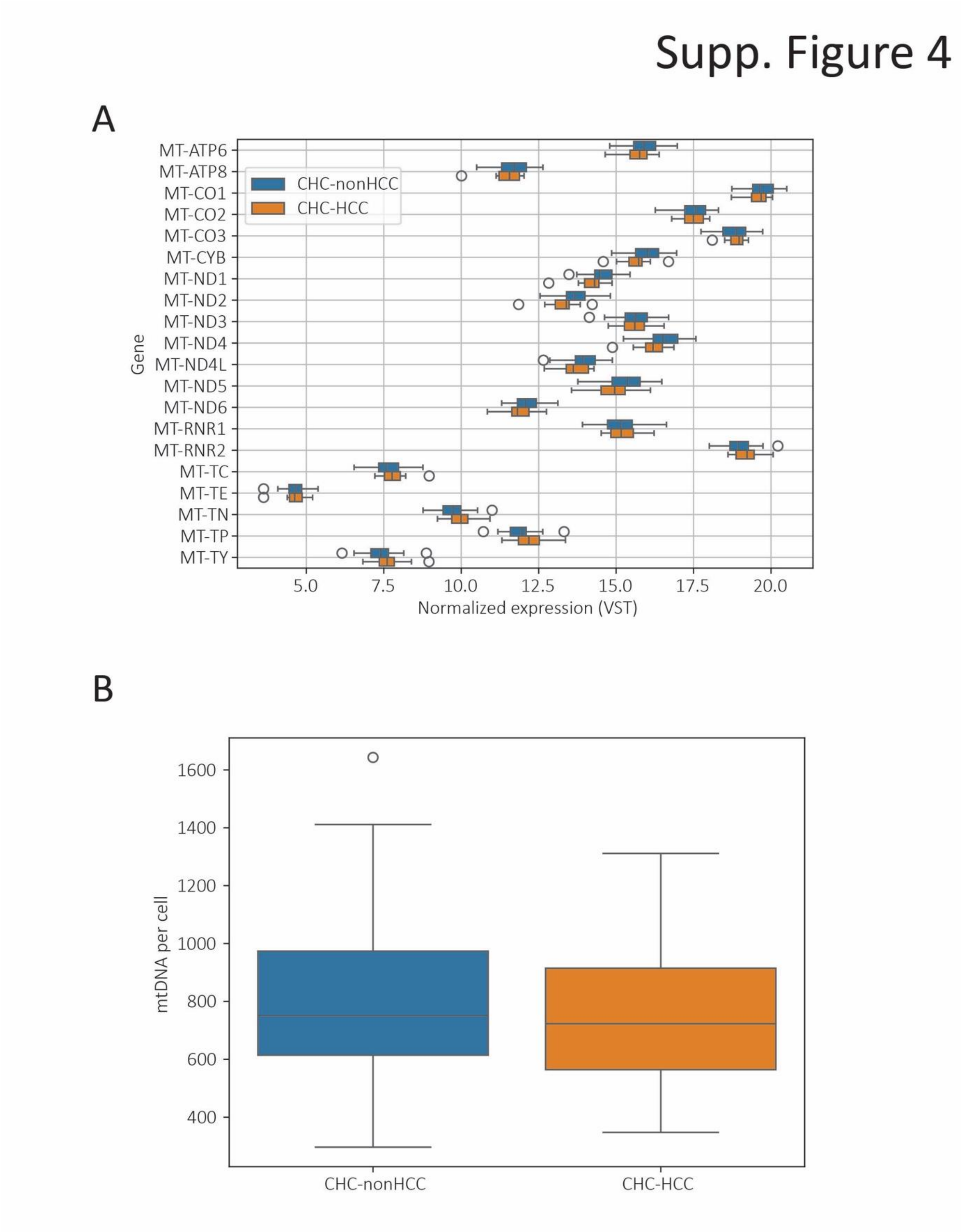

Fig. S4. Quantification of mitochondrial DNA levels by ddPCR.

A: Boxplots of all detected mitochondrial genes by RNAseq at baseline between *CHC-nonHCC* (blue) and *CHC-HCC* (orange). There was no statistically significant difference in abundence for any of the detected mitochondrial genes. Outliers are shown as open circles.

B: Mitochondrial DNA (mtDNA) levels per cell were measured using droplet digital PCR (ddPCR) as described in the Methods section and compared between *CHC-nonHCC* and *CHC-HCC* groups. Statistical analysis was performed using the Mann–Whitney U test. Outliers are shown as open circles.

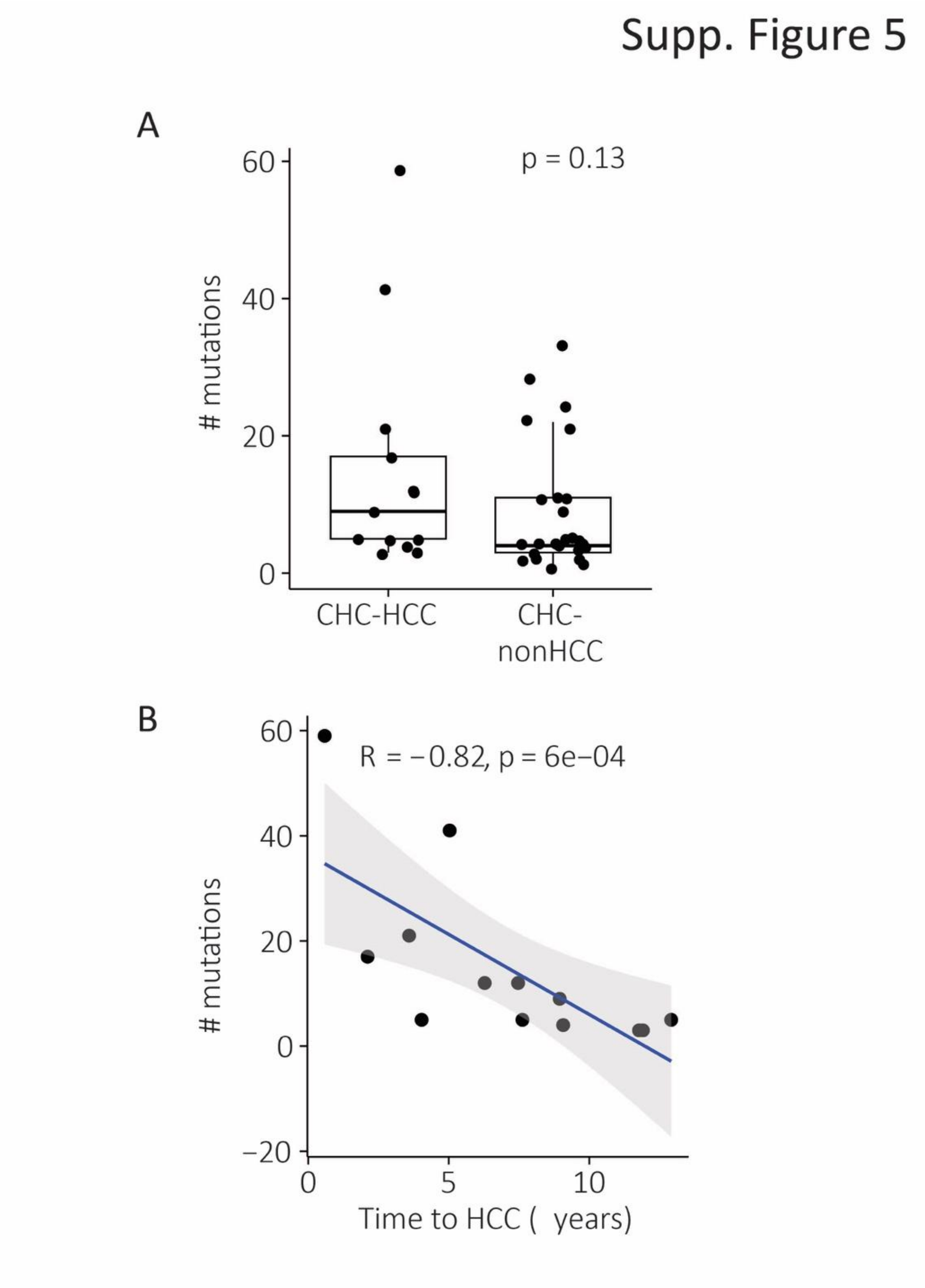

Fig. S5.

Number of somatic mutations in *CHC-HCC* and *CHC-nonHCC*.

A: Boxplots of the number of somatic mutations between *CHC-HCC* and *CHC-nonHCC*. Statistical comparison was assessed using Mann-Whitney U test. The boxes span the first and third quartiles, with the middle line indicating the medians.

B: Scatter plot of the number of somatic mutations against time to HCC diagnosis. Correlation assessed by the Spearman method. Blue line is the linear regression line and the gray shaded area encompasses the 95% confidence interval.

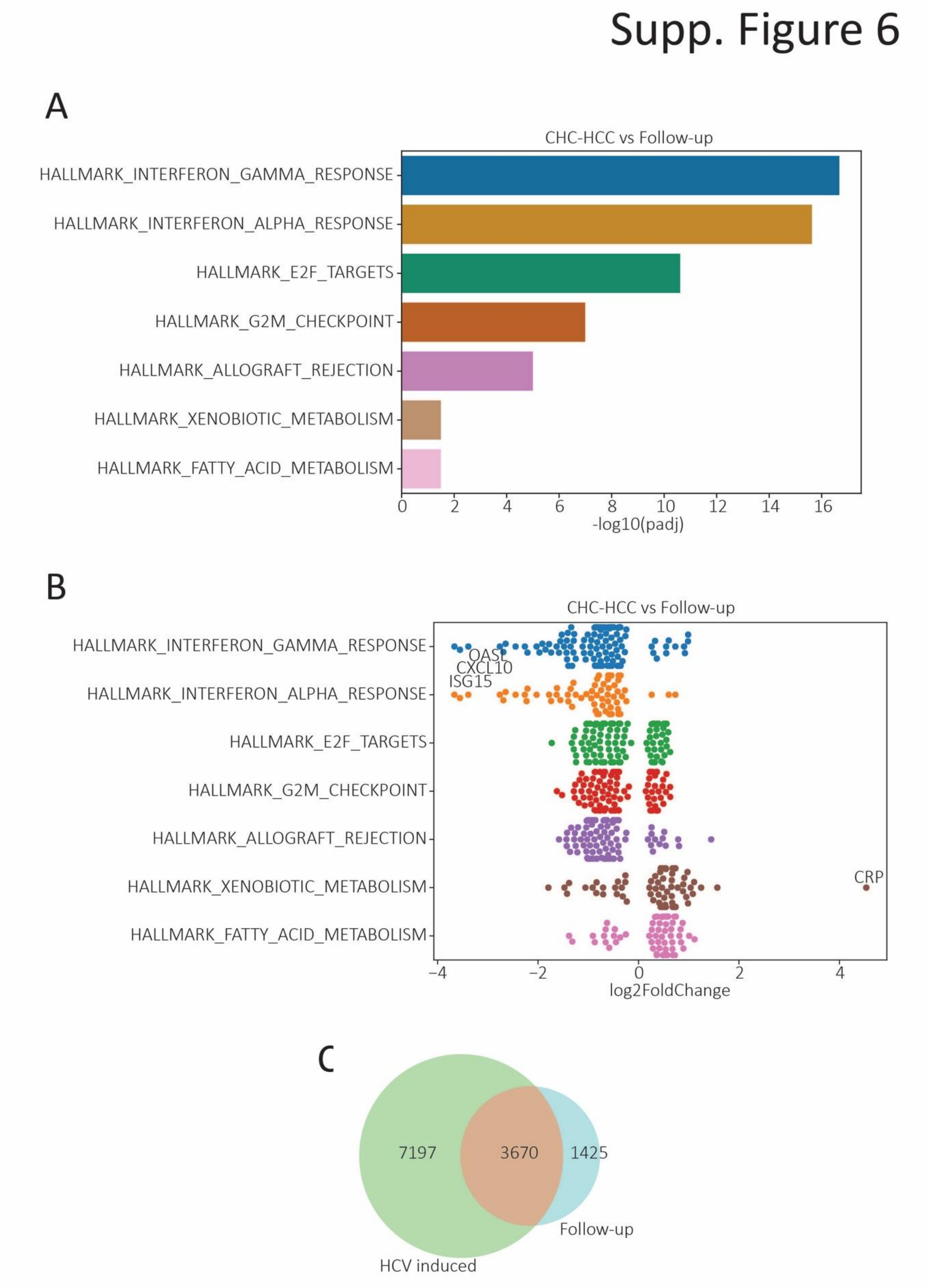

Fig. S6. Hallmark pathway enrichment analysis of differentially expressed genes (DEGs) between *CHC-HCC* and non-tumor follow-up biopsies.

A: Horizontal bar plot showing enriched Hallmark pathways among DEGs identified by transcriptomics of *CHC-HCC* and non-tumor follow-up liver biopsies using Fisher's Exact Test.

B: Swarm plot displaying the log2 fold change of all DEGs within each enriched Hallmark pathway. Each point represents a single gene, grouped by pathway.

C: Venn diagram. Circles indicate the sets of genes that are significantly differentially expressed (DEGs; FDR $\leq$ 0.05).

Green: CHC baseline biopsies with 15 normal livers with no HCV infection or other liver disease.

Blue: Paired comparison of CHC-HCC baseline biopsies with their matched non-tumor follow-up biopsies taken at time of HCC diagnosis.

Supp. Figure 7

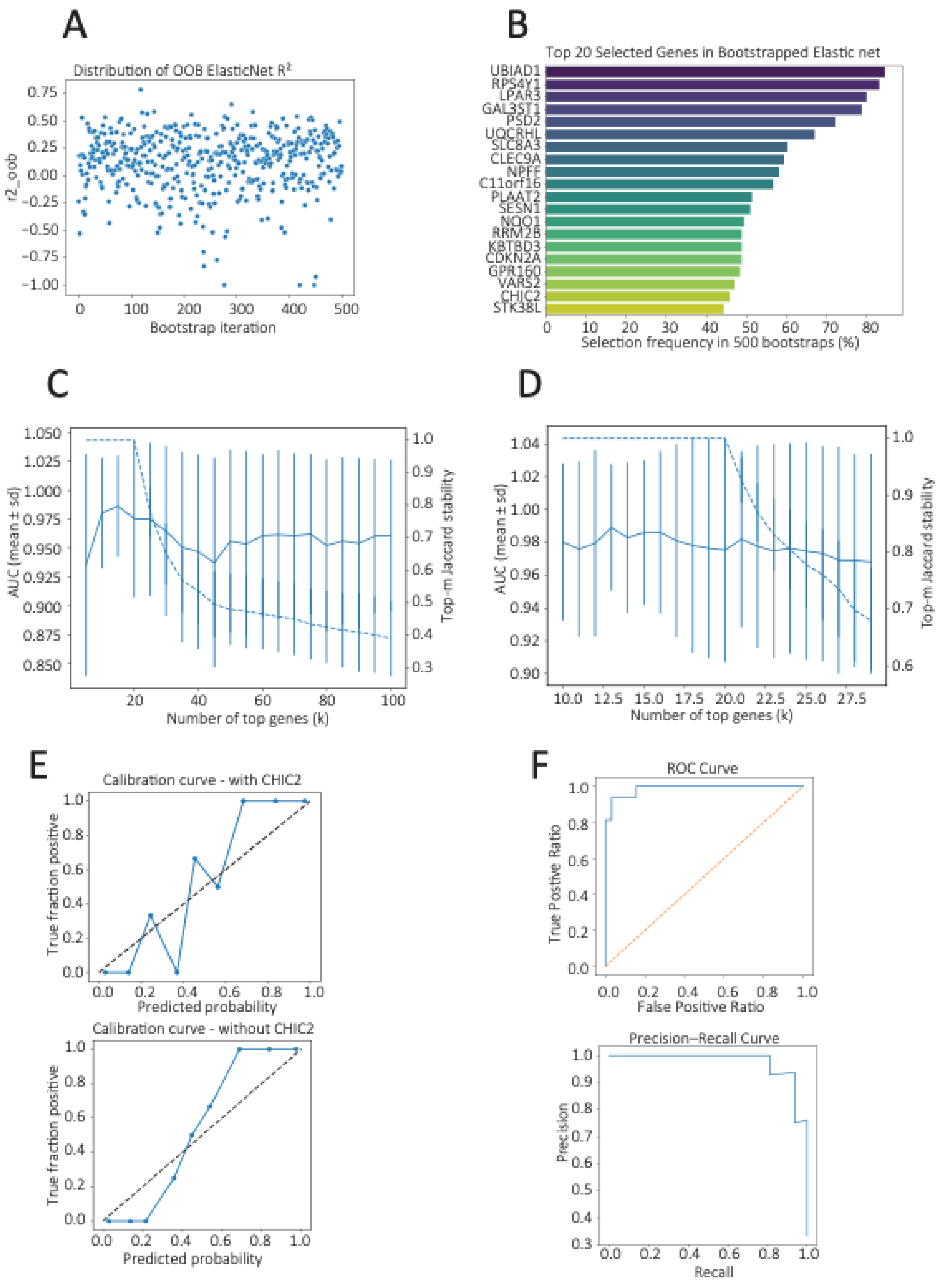

Fig. S7. Elastic net and linear regression prediction performance and feature selection.

A: Scatter plot of elastic net performance across 500 bootstrapped iterations. R^2 measures how much of the variance in the target variable is explained by the model. R^2 was recorded for out-of-bootstrap (OOB) test samples and plotted on the y-axis for each iteration on the x-axis.

B: Selection frequency of genes across 500 bootstrapped elastic net models shown as percent of bootstraps were the genes was selected (x-axis).

C: Mean classification performance (solid line, left y-axis) is shown as the area under the receiver operating characteristic curve (AUC) obtained from repeated stratified cross-validation, with error bars indicating standard deviation across folds. The dashed line (right y-axis) shows the Jaccard stability index of the top-m most important genes, quantifying the consistency of feature ranking across cross-validation runs. The x-axis indicates the number of top-ranked genes (k) included in the model from 5 to 100 genes with steps of additional 5 genes.

D: Same as C but with a more fine-tuned number of genes from 5 to 25 genes with steps of 1.

E: Calibration of the model was assessed by comparing predicted probabilities with observed outcome frequencies. Predicted risk for each sample was defined as the mean probability across 500 cross-validated models. Samples were grouped into 10 bins based on predicted probability, and the mean predicted probability (x-axis) was plotted against the observed fraction of positive cases within each bin (y-axis). The dashed diagonal line represents perfect calibration. Top panel: model including feature CHIC2. Bottom panel: model excluding feature CHIC2.

F: Model performance was evaluated using receiver operating characteristic (ROC) and precision–recall (PR) analyses based on predicted probabilities defined as the mean output across 500 cross-validated models.

Top panel: ROC curve showing the relationship between true positive rate (TPR) and false positive rate (FPR), with the dashed diagonal indicating random classifier performance.

Bottom panel: Precision–recall curve showing the relationship between precision and recall across prediction thresholds.

Table S1: Detailed patient characteristics
(linked file)

Table S2: List of significantly differentially expressed genes and their annotation to human reactome pathways.
(linked file)

Table S3: Reported connections between top differentially expressed genes and hepatocellular carcinoma

Gene Symbol	log2fold change	Reported connection to HCC	Reference(s)
SLITRK3	-3.335657387	-	
CYP2C19	-2.281460971	Downregulated in liver tissue of patients with HCC<br>Downregulation in HCC associated with aggressive tumor potential	[2]<br>[3]
SLC26A3	2.103507686	-	
MUC13	2.012032615	HCC initiation and tumor progression by activating Wnt signaling	[4]
C9orf57	1.963108429	-	
MISP	1.832217482	Overexpressed in sorafenib and lenvatinib resistant HCC cell lines	[5]
MYT1L	-1.827549987	Transcriptional repressor of WNT and NOTCH genes	[6]
ADCY1	-1.684218215	-	
LGI1	-1.508280419	-	
IL20RB	-1.459068245	-	
DCX	1.457842435	Marker of immature neuronal cells is overexpressed in HCC	[7]
KRT39	1.433241676	Transcriptional co-repressor promoting histone deacetylation and liver tumorigenesis	[8]
SLC30A2	1.376625657	-	
EFHD1	-1.364190655	Member of a 5 gene diagnostic biomarker for HCC	[9]
HOXD8	1.317224826	-	
AVPR1A	-1.312488931	Low expression associated with poor survival in HCC	[10]
PRR15	1.299491669	-	
RAB3B	1.293813597	Up-regulated in prognostically poor HCC	[11]
ANO4	1.263176288	-	
DPPA4	-1.243187966	-	

ASXL3	-1.242503332	-	
VXN	-1.241417189	-	
FAM3B	1.24005236	-	
USH2A	-1.234020079	-	
RAB25	1.20110019	Up-regulated in HCC and associated with advanced tumor stage and metastasis	[12]
GPC3	1.199048272	Surface marker of HCC	[13, 14]
ENSG00000278384	-1.196539061	-	
FAM237B	-1.17769491	-	
LPAR3	1.164247015	Overexpressed in hepatocellular carcinoma	[15]
CDX1	1.156514852	CDX3 expression associated with poor differentiation of HCC	[16]
BTNL8	1.135648647	-	
EPCAM	1.132857154	Biomarker of liver cancer stem cells	[17]
CTNNA3	-1.12790636	CTNNA3 is a tumor suppressor in HCC	[18]
TMPRSS11D	-1.121776515	-	
ACR	-1.121067287	-	
POF1B	1.103360334	Member of a multi-gene signature associated with vascular invasion of HCC	[19]
NQO1	1.092666912	Associated with aggressive HCC	[20]
EYA2	-1.092179012	Downregulated in HCC	[21]
ENSG00000284695	-1.080951241	-	
ENSG00000267561	-1.07654555	-	
CCDC70	-1.074652094		
NUTM2B	-1.074557682	NUTM2B-antisense lncRNA promotes HCC stemness feature	[22]
CST5	1.07201131	Overexpressed in HBV related HCC	[23]
PPP1R1A	-1.065562023	Downregulated in HCC	[24]
SLC45A2	-1.06547339	SLC45A2-AMACR fustion transcripts detected in serum of patients with HCC	[25]
CABCOCO1	1.055723015	-	
VWDE	-1.055325207	-	
MUC4	-1.050313605	-	
PLAAT2	1.045317223	-	
SDCBP2	1.037124704	-	
RGS13	1.036120453	-	
S100P	1.030553782	Upregulated in HCC	[26]
MMEL1	1.029364196	-	
PRICKLE4	-1.023659569	Negatively regulates Wnt/beta-catenin pathway in liver cancer	[27]

GRIN2A	-1.018892512	-

Table S4: List of somatic mutations identified in 13 *CHC-HCC* and 25 *CHC-nonHCC* samples.
(linked file)

Table S5: Clinical characterisics of normal liver biopsy cohort patients.

Biopsy ID	Sex	Age	Histology	Indication for liver biopsy
B885	m	63	normal	isolated GGT elevation
B927	m	60	normal	calcified liver lesion
B986	m	49	normal	isolated GGT elevation
C051a	f	62	normal	brest cancer metastasis
C173	m	61	normal	isolated GGT elevation
C222a	f	30	normal	FNH
C250a	m	80	normal	NET metastasis
C354a	f	42	normal	brest cancer metastasis
C369	m	36	normal	discrete drug induced liver injury
C445	m	33	normal	chronic portal vene thrombosis
C491	m	43	normal	isolated GGT elevation
C545	m	54	normal	unclear GGT- and AP-elevation
C686	f	53	normal	discrete drug induced liver injury
C712	m	46	normal	isolated GGT elevation
C782	m	77	normal	urothelial cancer metastasis
D568	f	56	normal	FNH
D605	m	73	normal	adrenal cancer metastasis
D635	m	44	normal	isolated GGT elevation
D738	f	29	normal	duodenal adenocarcinoma metastasis

Supplementary references

[1] Ng CKY, **Dazert E**, **Boldanova T**, et al. Integrative proteogenomic characterization of hepatocellular carcinoma across etiologies and stages. Nat Commun 2022;13:2436.
[2] Gallon J, Coto-Llerena M, Ercan C, et al. Epigenetic priming in chronic liver disease impacts the transcriptional and genetic landscapes of hepatocellular carcinoma. Mol Oncol 2022;16:665–682.
[3] Ashida R, Okamura Y, Ohshima K, et al. The down-regulation of the CYP2C19 gene is associated with aggressive tumor potential and the poorer recurrence-free survival of hepatocellular carcinoma. Oncotarget 2018;9:22058–22068.
[4] Dai Y, Liu L, Zeng T, et al. Overexpression of MUC13, a Poor Prognostic Predictor, Promotes Cell Growth by Activating Wnt Signaling in Hepatocellular Carcinoma. Am J Pathol 2018;188:378–391.
[5] Wang Z, Wu L, Zhou Y, et al. Protein and metabolic profiles of tyrosine kinase inhibitors co-resistant liver cancer cells. Front Pharmacol 2024;15:1394241.
[6] Weigel B, Tegethoff JF, Grieder SD, et al. MYT1L haploinsufficiency in human neurons and mice causes autism-associated phenotypes that can be reversed by genetic and pharmacologic intervention. Mol Psychiatry 2023;28:2122–2135.
[7] Hernandez CA, Verzeroli C, Roca-Suarez AA, et al. Hepatocellular carcinoma hosts cholinergic neural cells and tumoral hepatocytes harboring targetable muscarinic receptors. JHEP Rep 2025;7:101245.
[8] Han S, Fan H, Zhong G, et al. Nuclear KRT19 is a transcriptional corepressor promoting histone deacetylation and liver tumorigenesis. Hepatology 2025;81:808–822.
[9] Zheng H, Han X, Liu Q, et al. Construction of immune-related molecular diagnostic and predictive models of hepatocellular carcinoma based on machine learning. Heliyon 2024;10:e24854.
[10] Fekry B, Ribas-Latre A, Drunen RV, et al. Hepatic circadian and differentiation factors control liver susceptibility for fatty liver disease and tumorigenesis. FASEB J 2022;36:e22482.
[11] Tsunedomi R, Yoshimura K, Kimura Y, et al. Elevated expression of RAB3B plays important roles in chemoresistance and metastatic potential of hepatoma cells. BMC Cancer 2022;22:260.
[12] Geng D, Zhao W, Feng Y, et al. Overexpression of Rab25 promotes hepatocellular carcinoma cell proliferation and invasion. Tumour Biol 2016;37:7713–7718.
[13] Baumhoer D, Tornillo L, Stadlmann S, et al. Glypican 3 expression in human nonneoplastic, preneoplastic, and neoplastic tissues: a tissue microarray analysis of 4,387 tissue samples. Am J Clin Pathol 2008;129:899–906.
[14] Stadlmann S, Gueth U, Baumhoer D, et al. Glypican-3 expression in primary and recurrent ovarian carcinomas. Int J Gynecol Pathol 2007;26:341–344.
[15] Zuckerman V, Sokolov E, Swet JH, et al. Expression and function of lysophosphatidic acid receptors (LPARs) 1 and 3 in human hepatic cancer progenitor cells. Oncotarget 2016;7:2951–2967.
[16] Zheng H, Yang Y, Wang MC, et al. Low CDX1 expression predicts a poor prognosis for hepatocellular carcinoma patients after hepatectomy. Surg Oncol 2016;25:171–177.
[17] Yamashita T, Ji J, Budhu A, et al. EpCAM-positive hepatocellular carcinoma cells are tumor-initiating cells with stem/progenitor cell features. Gastroenterology 2009;136:1012–1024.

[18] He B, Li T, Guan L, et al. CTNNA3 is a tumor suppressor in hepatocellular carcinomas and is inhibited by miR-425. Oncotarget 2016;7:8078–8089.
[19] Yi B, Tang C, Tao Y, et al. Definition of a novel vascular invasion-associated multi-gene signature for predicting survival in patients with hepatocellular carcinoma. Oncol Lett 2020;19:147–158.
[20] Yang Y, Zheng J, Wang M, et al. NQO1 promotes an aggressive phenotype in hepatocellular carcinoma via amplifying ERK-NRF2 signaling. Cancer Sci 2021;112:641–654.
[21] Liu ZK, Li C, Zhang RY, et al. EYA2 suppresses the progression of hepatocellular carcinoma via SOCS3-mediated blockade of JAK/STAT signaling. Mol Cancer 2021;20:79.
[22] Li W, Zeng M, Ning Y, et al. m(6)A-Methylated NUTM2B-AS1 Promotes Hepatocellular Carcinoma Stemness Feature via Epigenetically Activating BMPR1A Transcription. J Hepatocell Carcinoma 2024;11:2393–2411.
[23] Zhou X, Wang X, Huang K, et al. Investigation of the clinical significance and prospective molecular mechanisms of cystatin genes in patients with hepatitis B virus-related hepatocellular carcinoma. Oncol Rep 2019;42:189–201.
[24] Wu X, Wang Y, Yang M, et al. Exploring prognostic value and regulation network of PPP1R1A in hepatocellular carcinoma. Hum Cell 2022;35:1856–1868.
[25] Yu YP, Tsung A, Liu S, et al. Detection of fusion transcripts in the serum samples of patients with hepatocellular carcinoma. Oncotarget 2019;10:3352–3360.
[26] Yuan RH, Chang KT, Chen YL, et al. S100P expression is a novel prognostic factor in hepatocellular carcinoma and predicts survival in patients with high tumor stage or early recurrent tumors. PLoS One 2013;8:e65501.
[27] Chan DW, Chan CY, Yam JW, et al. Prickle-1 negatively regulates Wnt/beta-catenin pathway by promoting Dishevelled ubiquitination/degradation in liver cancer. Gastroenterology 2006;131:1218–1227.
