# Supplementary material for: Hepatitis C virus can induce gene expression changes associated with hepatocarcinogenesis
Source: JHEP Rep. 2026 May 29;8(8):101897. doi: 10.1016/j.jhepr.2026.101897 (PMC13380720; doi:10.1016/j.jhepr.2026.101897)
Supplement: Multimedia compoent 2 [file mmc2.docx]

**JHEP Reports**

**CTAT methods**

Tables for a “Complete, Transparent, Accurate and Timely account” (CTAT) are now mandatory for all revised submissions. The aim is to enhance the reproducibility of methods.

- Only include the parts relevant to your study
- Refer to the CTAT in the main text as ‘Supplementary CTAT Table’
- Do not add subheadings
- Add as many rows as needed to include all information
- Only include one item per row

**If the CTAT form is not relevant to your study, please outline the reasons why:**

|  |
| --- |

- 1. **Antibodies**

| **Name** | **Citation** | **Supplier** | **Cat no.** | **Clone no.** |
| --- | --- | --- | --- | --- |
| Ki-67 |  | DAKO | AB_2890068 (IR626) | IR626 / Ready-to-use; Ventana Benchmark Ultra |
| c-MYC |  | Roche | 06504612001 (Y69) | Y69 / Ready-to-use; Ventana Benchmark Ultra |

- 1. **Cell lines**

| **Name** | **Citation** | **Supplier** | **Cat no.** | **Passage no.** | **Authentication test method** |
| --- | --- | --- | --- | --- | --- |
| Not applicable |  |  |  |  |  |

- 1. **Organisms**

| **Name** | **Citation** | **Supplier** | **Strain** | **Sex** | **Age** | **Overall n number** |
| --- | --- | --- | --- | --- | --- | --- |
| Homo sapiens |  | University Hospital Basel |  | Mixed | Adult | 48 |

- 1. **Sequence based reagents**

| **Name** | **Sequence** | **Supplier** |
| --- | --- | --- |
| Mitochondrial DNA primers (forward) | 5’- TCCTAATGCTTACCGAACGA -3’ | Custom |
| Mitochondrial DNA primers (reverse) | 5’- GCGTCAGCGAAGGGTTGTAG -3’ | Custom |
| IMAP assay primers (forward) | 5’- TTTTCAGCTCCCAAGTGTCC -3’ | Custom |
| IMAP assay primers (reverse) | 5’- GCCGAGAGCAGGTAGCAGT -3’ | Custom |

- 1. **Biological samples**

| **Description** | **Source** | **Identifier** |
| --- | --- | --- |
| Human liver biopsies (baseline CHC-HCC n=16; baseline CHC-nonHCC n=32) | University Hospital Basel liver biopsy biobank | Ethics: EKNZ 2014-099 |
| Matched tumor and non-tumor liver biopsies at time of HCC diagnosis (CHC-HCC) | University Hospital Basel | — |
| Matched blood samples (germline control) | University Hospital Basel | — |
| Normal HCV-negative liver biopsies (n=15) | University Hospital Basel / prior cohort | — |

- 1. **Deposited data**

| **Name of repository** | **Identifier** | **Link** |
| --- | --- | --- |
| European Genome-phenome Archive (EGA) | EGAS50000001096 | https://ega-archive.org |
| European Genome-phenome Archive (EGA) | EGAS50000001097 | https://ega-archive.org |
| European Genome-phenome Archive (EGA) | EGAS50000001098 | https://ega-archive.org |

- 1. **Software**

| **Software name** | **Manufacturer** | **Version** |
| --- | --- | --- |
| STAR | https://github.com/alexdobin/STAR | v2.7.9a |
| Trimmomatic | http://www.usadellab.org/cms/?page=trimmomatic | v0.39 |
| FeatureCounts (Subread package) | http://subread.sourceforge.net/ | Version NOT PROVIDED |
| pyDESeq2 | https://pydeseq2.readthedocs.io/ | v0.5.3 |
| Python | https://www.python.org/ | v3.14.0 |
| SciPy | https://scipy.org/ | v1.6.13 |
| biomaRt | https://bioconductor.org/packages/biomaRt | v2.54.1 |
| ImSig | https://bioconductor.org/packages/ImSig | v1.1.3 |
| GSEApy | https://gseapy.readthedocs.io/ | v1.1.5 |
| Enrichr | https://maayanlab.cloud/Enrichr/ | Web service / API |
| MSigDB | https://www.gsea-msigdb.org/gsea/msigdb | v2024.1.Hs |
| BWA | https://github.com/lh3/bwa | v0.7.17 |
| GATK | https://gatk.broadinstitute.org/ | v3.6 |
| Picard | http://broadinstitute.github.io/picard/ | v2.25.2 |
| Strelka2 | https://github.com/Illumina/strelka | v2.9.10 |
| MuSE | https://github.com/ding-lab/muse | v2.0.4 |
| cgpCaVEManWrapper | https://github.com/cancerit/cgpCaVEManWrapper | v1.18.3 |
| MuTect2 CreateSomaticPanelOfNormals | GATK | See GATK / MuTect2 |
| R | https://www.r-project.org/ | v4.0.3 |
| scikit-learn | https://scikit-learn.org/ | v1.7.0 |

- 1. **Other (*e.g*. drugs, proteins, vectors etc.)**

|  |  |  |
| --- | --- | --- |

- 1. **Please provide the details of the corresponding methods author for the manuscript:**

| Markus H. Heim, 1Hepatology Research Group, Department of Biomedicine, University of Basel, Switzerland, markus.heim@unibas.ch, tel +41 61 777 74 90 |
| --- |

**2.0 Please confirm for randomised controlled trials all versions of the clinical protocol are included in the submission. These will be published online as supplementary information.**

|  |
| --- |
